# Nitric Oxide Sensing in Plants Is Mediated by Proteolytic Control of Group VII ERF Transcription Factors

**DOI:** 10.1016/j.molcel.2013.12.020

**Published:** 2014-02-06

**Authors:** Daniel J. Gibbs, Nurulhikma Md Isa, Mahsa Movahedi, Jorge Lozano-Juste, Guillermina M. Mendiondo, Sophie Berckhan, Nora Marín-de la Rosa, Jorge Vicente Conde, Cristina Sousa Correia, Simon P. Pearce, George W. Bassel, Bulut Hamali, Prabhavathi Talloji, Daniel F.A. Tomé, Alberto Coego, Jim Beynon, David Alabadí, Andreas Bachmair, José León, Julie E. Gray, Frederica L. Theodoulou, Michael J. Holdsworth

**Affiliations:** 1 Division of Plant and Crop Sciences, School of Biosciences, University of Nottingham, Loughborough LE12 5RD, UK; 2 Department of Molecular Biology and Biotechnology, University of Sheffield, Sheffield S10 2TN, UK; 3 Instituto de Biología Molecular y Celular de Plantas, Consejo Superior de Investigaciones Científicas-Universidad Politécnica de Valencia, Ciudad Politécnica de la Innovación, 46022 Valencia, Spain; 4 Department of Biochemistry and Cell Biology, Max F. Perutz Laboratories, University of Vienna, Dr. Bohr Gasse 9, Vienna 1030, Austria; 5 School of Life Sciences, University of Warwick, Coventry CV4 7AL, UK; 6 Biological Chemistry and Crop Protection Department, Rothamsted Research, Harpenden AL5 2JQ, UK

## Abstract

Nitric oxide (NO) is an important signaling compound in prokaryotes and eukaryotes. In plants, NO regulates critical developmental transitions and stress responses. Here, we identify a mechanism for NO sensing that coordinates responses throughout development based on targeted degradation of plant-specific transcriptional regulators, the group VII ethylene response factors (ERFs). We show that the N-end rule pathway of targeted proteolysis targets these proteins for destruction in the presence of NO, and we establish them as critical regulators of diverse NO-regulated processes, including seed germination, stomatal closure, and hypocotyl elongation. Furthermore, we define the molecular mechanism for NO control of germination and crosstalk with abscisic acid (ABA) signaling through ERF-regulated expression of *ABSCISIC ACID INSENSITIVE5* (*ABI5*). Our work demonstrates how NO sensing is integrated across multiple physiological processes by direct modulation of transcription factor stability and identifies group VII ERFs as central hubs for the perception of gaseous signals in plants.

## Highlights

• We elucidate a general molecular mechanism for nitric oxide sensing in plants
• Group VII ERFs act as nitric oxide sensors via the N-end rule pathway
• Group VII ERFs are shown to mediate crosstalk between nitric oxide and abscisic acid
• The N-end rule regulates nitric oxide homeostasis through group VII ERFs

## Graphical Abstract

**Figure:**
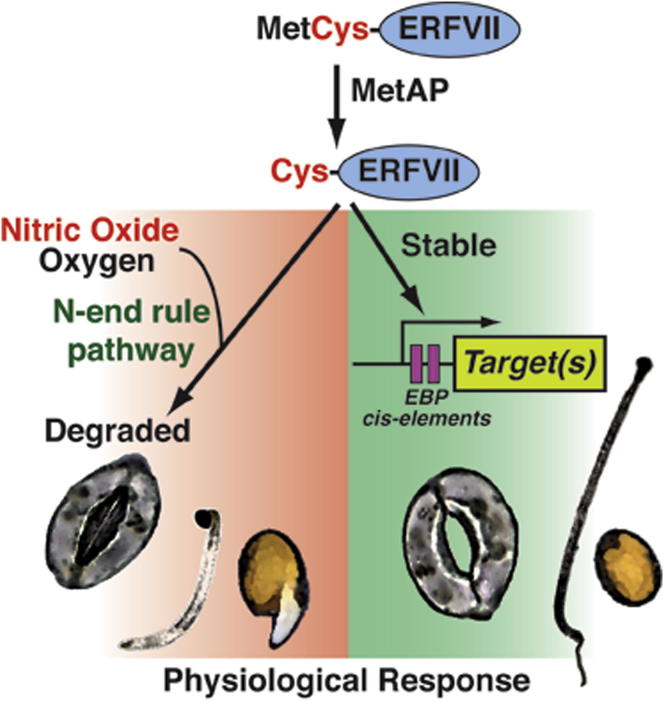

## Teaser

Gibbs et al. report that NO-dependent degradation of group VII ERF transcription factors by the N-end rule pathway mediates NO sensing and signal transduction, identifying these transcription factors as central hubs for the perception of gaseous signals in plants.

## Introduction

Nitric oxide (NO) is a small gaseous molecule that functions as an important developmental signal in prokaryotes and eukaryotes. In plants, NO regulates many different processes throughout development, including seed dormancy, postgerminative vegetative growth, flowering, stomatal aperture, leaf senescence, and response to pathogens (Mur et al., 2013). In mammals, endogenous production of NO occurs predominantly through the activity of nitric oxide synthase (NOS) isoforms. However, plant genomes do not contain NOS homologs, and despite its importance as a signaling molecule, the origins of NO in plant cells are poorly understood (Gupta et al., 2011a). The majority of NO synthesized in plants is thought to be derived from two unrelated enzyme-based pathways, one involving two functionally redundant nitrate reductases (NIAs) and the other requiring the undefined action of nitric oxide-associated protein 1 (AtNOA-1). Due to the highly reactive nature of NO, it has been proposed that it is unlikely to interact with a single defined receptor (Besson-Bard et al., 2008). Although NO-dependent protein modifications, such as *S*-nitrosylation, Y nitration, and metal nitrosylation, have been identified for specific regulatory proteins (Gupta et al., 2011b; Kovacs and Lindermayr, 2013; Lozano-Juste et al., 2011), no general mechanism that coordinates NO sensing across multiple developmental processes has been identified previously in plants.

**Figure 1:**
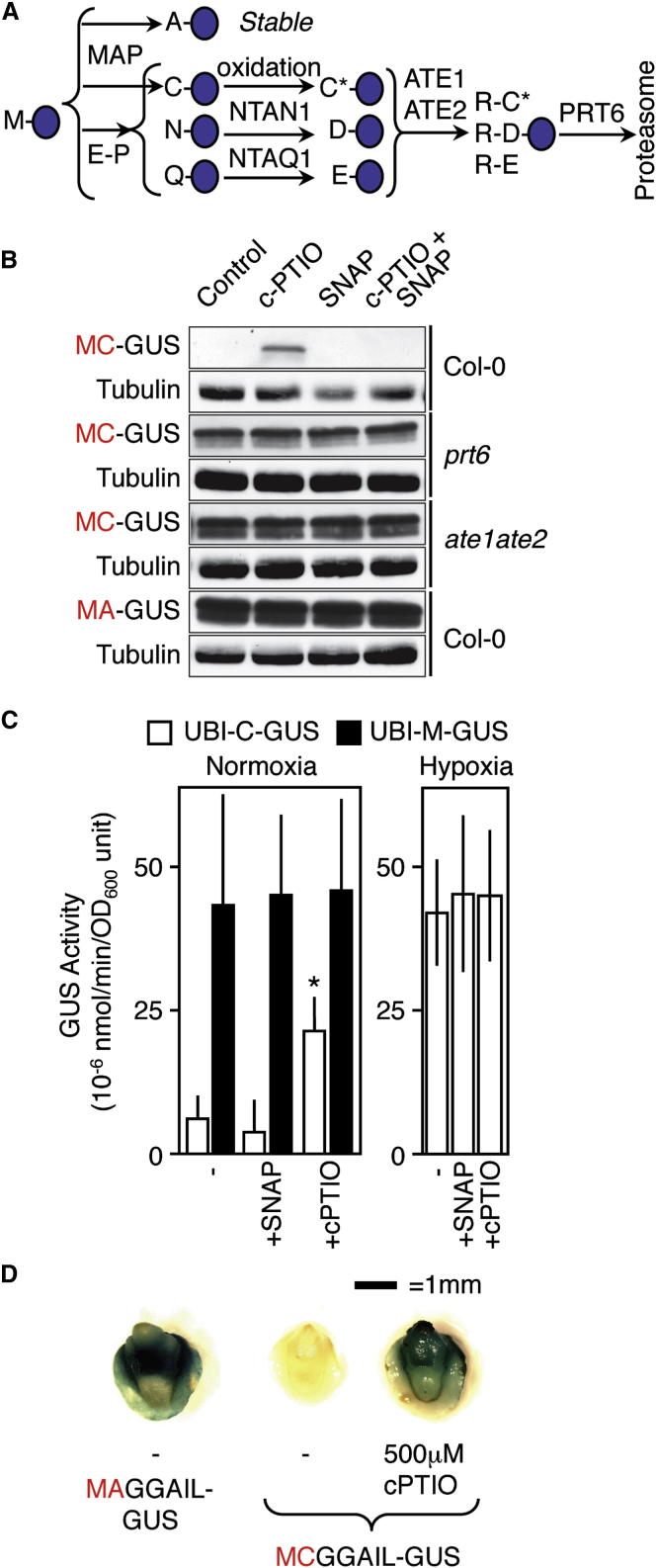
The Cys-Arg/N-End Rule Pathway Acts as a Nitric Oxide Sensor in Plants (A) Schematic of the relationship between methionine aminopeptidase (MAP) activity and the Cys-Arg/N-end rule pathway. PRT6, PROTEOLYSIS6 E3 ligase; ATE, arginyl tRNA transferase; NTAN1, asparagine-specific N-terminal amidase; NTAQ1, glutamine-specific N-terminal amidase; E-P, endopeptidase. Amino acids are indicated with single letter codes; C^∗^ = oxidized cysteine. The action of MAP on MA-initiating proteins results in a stabilizing Nt residue. (B) Western blot of total seedling protein showing MC/MA-GUS stability in response to cPTIO or SNAP in WT (Col-0) and N-end rule mutant seedlings. MC-GUS stability is enhanced by cPTIO in WT, but not in *prt6* or *ate1ate2*. MA-GUS is constitutively stable regardless of NO availability. (C) GUS enzyme activity of UBI-M/C-GUS in response to SNAP or cPTIO for normoxic and hypoxia-treated samples. cPTIO enhances C-GUS activity under normoxia, but neither cPTIO nor SNAP affect the enhanced C-GUS activity observed under hypoxia. M-GUS activity is unaffected by NO treatments. Error bars denote SE. ^∗^p < 0.05. (D) GUS staining of transgenic barley embryos expressing M(C/A)-GGAIL-GUS (GUS initiating at the N terminus with either MCGGAIL or MAGGAIL) in response to cPTIO application. MCGGAIL-GUS protein is stabilized in the presence of cPTIO. See also Figures S1 and S2.

The N-end rule pathway of targeted proteolysis relates the stability of a protein to the nature of its N-terminal (Nt) residue (Varshavsky, 2011). This pathway is highly conserved in eukaryotes and plays a key role in the regulation of many growth and developmental processes, including apoptosis, cardiovascular development, DNA replication, and response to abiotic stresses (Sriram et al., 2011). There are two characterized branches of the N-end rule pathway: the Ac/N-end rule pathway, which targets proteins with N-terminally acetylated (Ac) residues, and the Arg/N-end rule, which recognizes specific unacetylated Nt residues (Sriram et al., 2011). Eukaryotic proteins are synthesized with methionine (Met) at the N terminus, but new N termini can be generated via the action of endopeptidases or by cotranslational cleavage of Nt-Met by methionine aminopeptidases (MAPs). Newly exposed Nt residues may be stabilizing or destabilizing; proteins containing destabilizing residues (N-degrons) are ubiquitinated by specific E3 ligases (N-recognins) and targeted for proteosomal degradation. N-degrons can also be created through enzymatic or chemical modification of the N-terminal amino acid (Figure 1A). For example, in the cysteine (Cys) subdivision of the Arg/N-end rule pathway (Cys-Arg/N-end rule), exposed N-terminal Cys residues are susceptible to oxidation, which permits subsequent arginylation by Arg-tRNA protein transferases (ATEs; Figure 1A), followed by ubiquitination by N-recognins that recognize the Arg destabilizing residue.

In *Arabidopsis thaliana*, PROTEOLYSIS6 (PRT6) is the N-recognin for the Arg/N-end rule pathway, and there are two ATE isoforms (ATE1 and ATE2) (Garzón et al., 2007; Graciet et al., 2009; Holman et al., 2009). The Arg/N-end rule pathway has several known functions in *Arabidopsis*, including the regulation of seed germination and subsequent seedling establishment through oil body breakdown, control of leaf and shoot development, and leaf senescence (Graciet et al., 2009; Holman et al., 2009; Yoshida et al., 2002). Recently, the Cys subdivision of the Arg/N-end rule pathway was shown to regulate oxygen sensing in plants by controlling the stability of the plant-specific group VII ERF transcription factors, which represent the first known physiological substrates of the plant N-end rule pathway (Gibbs et al., 2011; Licausi et al., 2011). Group VII ERFs are characterized by a conserved N-terminal domain initiating with the residues Met-Cys (MC), and in *Arabidopsis* there are five family members: HYPOXIA RESPONSIVE ERF 1 (HRE1), HRE2, RELATED TO AP 2.12 (RAP2.12), RAP2.2, and RAP2.3 (Nakano et al., 2006). These proteins are destabilized in the presence of oxygen, via oxidation of Cys2 following constitutive removal of Nt-Met by MAP activity (Figure 1A), but accumulate under low oxygen conditions and thus act as homeostatic sensors of hypoxia in plants (Gibbs et al., 2011; Licausi et al., 2011). In mice, destabilization of several MC-initiating REGULATOR OF G-PROTEIN SIGNALING (RGS) protein substrates involved in the regulation of cardiovascular development was shown to require NO in addition to oxygen in order to convert Nt-Cys to Cys sulfonic acid (Cys-SO_3_H) and permit arginylation by ATE (Hu et al., 2005; Jaba et al., 2013). This indicates that substrates of the Cys-Arg/N-end rule have the capacity to act as NO sensors.

In this study, we identify a unifying mechanism for NO sensing in plants based on targeted proteolysis of plant-specific transcriptional regulators. We show that group VII ERF transcription factors are sensors of NO via the N-end rule pathway and that this sensing coordinately regulates NO-mediated processes during growth and development. We show that reducing NO levels genetically by removing nitrate reductase activity restores constitutive stability of the group VII ERFs, suggesting that a single mechanism of NO synthesis may predominate in plant NO signaling. For one NO-regulated process, seed germination, we completely define the molecular mechanism from signal sensing by group VII ERFs through to the regulation of a key downstream transcription factor, ABSCISIC ACID INSENSITIVE 5 (ABI5); in doing so, we also uncover a mechanism of hormonal crosstalk between NO and ABA.

## Results

### Group VII ERF Transcription Factors Function as Nitric Oxide Sensors via the N-End Rule Pathway in Plants

To investigate a potential role for the Cys-Arg/N-end rule in NO sensing in plants, we first analyzed the in vivo stability of artificial N-end rule substrate reporters. These consisted of Cys N-degrons fused to β-glucuronidase (MC-GUS or UBI-C-GUS, which are modified GUS proteins bearing a Met-Cys N terminus or an N-terminal Ubiquitin-Cys fusion, respectively; see Figure S1 available online for further details), or the equivalent proteins with Cys substituted with the stabilizing residues Ala or Met (MA-GUS or UBI-M-GUS) (Figures 1 and S1). Treatment of WT (Col-0) seedlings with the cell-permeant NO scavenger 2-(4-carboxyphenyl)-4,4,5,5-tetramethylimidazoline-1-oxyl-3-oxide (cPTIO) stabilized GUS derived from MC or UBI-C proteins, an effect that was reversed when the NO donors S-nitroso-N-acetyl-DL-penicillamine (SNAP) or sodium nitroprusside (SNP) were also present. However, cPTIO did not enhance MA-GUS or UBI-M-GUS levels (Figures 1B, 1C, and S2). Stability was not influenced by cPTIO or NO donors in either *prt6* or *ate1ate2*, mutants which lack Arg/N-end rule components, and reporter transcript levels were unaffected by all treatments (Figures 1B and S2). Similar effects of cPTIO on protein stability were obtained in transgenic barley expressing M(C/A)GGAIL-GUS (containing WT or mutated N termini of barley group VII ERFs), indicating that this mechanism is conserved across flowering plants (Figure 1D).

**Figure 2:**
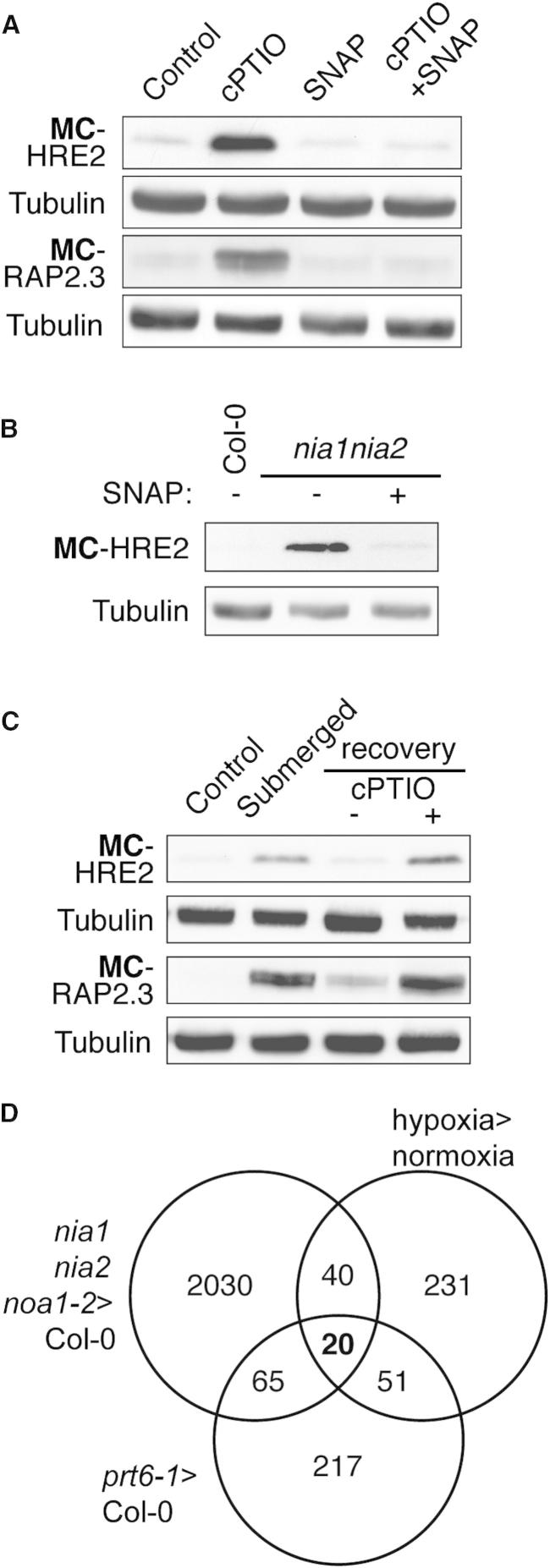
Nitric Oxide Regulates Group VII ERF Stability and the Hypoxia Transcriptome (A) Western blot of total seedling protein showing in vivo stability of HRE2-HA and RAP2.3-HA in seedlings treated with cPTIO or SNAP. (B) Western blot of total seedling protein showing enhanced MC-HRE2-HA stability in the NO-deficient *nia1nia2* mutant with or without NO donor SNAP. (C) Stability of HRE2-HA and RAP2.3-HA in submergence-induced hypoxia, and recovery in normoxia ± cPTIO. Reintroduction of oxygen following hypoxia is insufficient to destabilize ERFs in the absence of NO. (D) Venn diagram showing the overlap in upregulated genes between *prt6* > Col-0, *nia1nia2noa1-2* > Col-0, and Col-0: hypoxia > normoxia. All experiments were carried out with seedlings. See also Figure S2.

Next, we tested the effect of NO on the stability of two representative physiological N-end rule substrates, the MC-initiating group VII ERFs RAP2.3 and HRE2, which are stabilized under hypoxia (Gibbs et al., 2011). MC-RAP2.3-HA and MC-HRE2-HA accumulated in WT seedlings treated with cPTIO (Figures 2A and S2). Mutant MA-initiating versions of each protein in WT were constitutively stable regardless of NO availability, as was MC-HRE2-HA in the *prt6* background. To confirm the requirement for NO in group VII ERF degradation, we crossed *35S:MC-HRE2-HA* into the nitrate reductase (NR)-deficient mutant *nia1nia2*, which has highly reduced NO levels (Desikan et al., 2002; Lozano-Juste and León, 2010; Rockel et al., 2002). Protein stability was markedly enhanced in this background and reduced in the presence of SNAP (Figure 2B). These experiments suggest that group VII ERFs function as sensors for NO in plants and indicate a major role for NR-derived NO in the destabilization of Nt-Cys N-end rule substrates.

### Group VII ERFs Act as Hubs for the Perception and Transduction of Both Oxygen and Nitric Oxide Signals

Since group VII ERFs had previously been shown to accumulate under hypoxia, we next examined the relative contributions of both oxygen and NO to the degradation of Nt-Cys-initiating N-end rule substrates. MC-RAP2.3-HA and MC-HRE2-HA were stabilized when seedlings were grown under hypoxia, and cPTIO prevented their degradation following subsequent transfer to normoxia (Figure 2C). Furthermore, for the UBI-C-GUS construct, while GUS activity was increased under hypoxia, no effect on activity was observed when cPTIO or SNAP was supplied since, in this case, the C-GUS is already stabilized by lack of oxygen (Figure 1C). Therefore, these data indicate a requirement for both NO and oxygen for Nt-Cys oxidation and protein destabilization in plants and suggest that the group VII ERFs may act as hubs for the perception of both gases. In light of this, we examined the relationships between genome expression controlled by NO, hypoxia, and the N-end rule pathway. As the NO scavenging effect of cPTIO is transitory, we analyzed the influence of mutants with reduced NO levels (Lozano-Juste and León, 2010). We compared transcriptome data from seedlings of Arg/N-end rule pathway mutants (*prt6* and *ate1ate2*) (Gibbs et al., 2011), NO-deficient mutants (*noa1-2*, *nia1nia2*, and *noa1-2nia1nia2*), and WT under hypoxia (Gibbs et al., 2011). A highly significant overlap (hypergeometric test; Figure 2D, Table S1) of induced gene expression was identified in all treatments, including key members of the hypoxia-induced “core 49” gene set (Mustroph et al., 2009), such as *ALCOHOL DEHYDROGENASE1* (*ADH1*) (Chung and Ferl, 1999). These data suggest that the plant transcriptional response to hypoxia is regulated by endogenous NO levels in addition to oxygen availability and that both gases can influence gene expression through the N-end rule pathway.

### Nitric Oxide Controls Germination through Group VII ERFs

**Figure 3:**
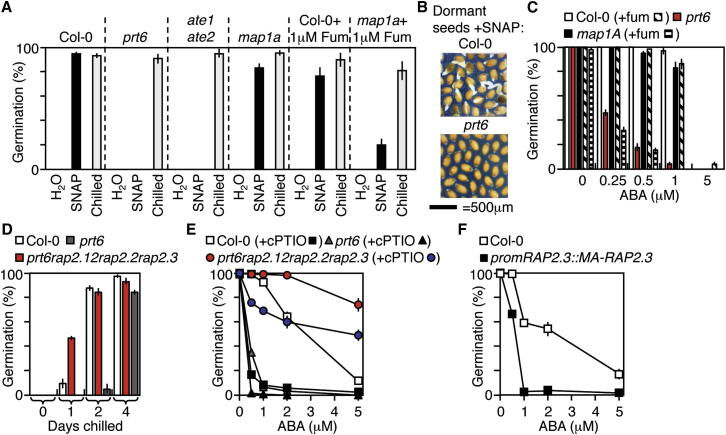
Nitric Oxide Regulates Seed Germination through the N-End Rule Pathway (A) Germination response of dormant seeds to SNAP and chilling. WT (Col-0) dormant seeds germinate in response to SNAP, whereas *prt6* and *ate1ate2* are insensitive, and lines with reduced MAP activity (*map1a* + fumagillin; Fum) have significantly reduced responsiveness. Chilling controls are included to show seed viability. (B) Pictures of seeds after 7 days imbibition in the presence of SNAP. (C) Germination ABA sensitivity of *map1A* seeds with or without fumagillin (Fum) relative to WT and *prt6*, showing that abolishing MAP activity enhances seed ABA hypersensitivity (similar to *prt6*). (D) Seed chilling sensitivity of *prt6rap2.12rap2.2rap2.3*. *prt6* seeds are more dormant than WT (requiring longer chilling to break dormancy), whereas *prt6rap2.12rap2.2rap2.3* seeds are less dormant. (E) ABA sensitivity of *prt6rap2.12rap2.2rap2.3* seeds with or without cPTIO. *prt6rap2.12rap2.2rap2.3* has strongly reduced sensitivity to ABA relative to *prt6*. cPTIO-mediated enhancement of ABA sensitivity is also greatly reduced in *prt6rap2.12rap2.2rap2.3*. (F) ABA sensitivity of *promRAP2.3::MA-RAP2.3* seeds. Seeds expressing a dominant-stable MA-RAP2.3 protein driven from its own promoter are ABA hypersensitive, similar to *prt6*. Error bars denote SE. See also Figures S3 and S4.

A wide range of plant developmental processes and responses to abiotic and biotic stresses are regulated by NO (Mur et al., 2013), including the alleviation of seed dormancy, inhibition of hypocotyl elongation, and enhancement of stomatal closure (Bethke et al., 2007; Desikan et al., 2002; Lozano-Juste and León, 2010). In none of these cases is the molecular mechanism of NO sensing known; therefore, we examined a potential role for the N-end rule pathway in controlling these responses. Seed dormancy is a key agronomic and ecological trait removed by after-ripening of dry seeds or chilling of imbibed seeds (Holdsworth et al., 2008b). NO activates germination by promoting dormancy release and reducing sensitivity to ABA, a positive regulator of seed dormancy. The endosperm layer surrounding the embryo maintains dormancy and responds to NO (Bethke et al., 2004, 2007; Liu et al., 2009). Cell separation at the micropylar endosperm (adjacent to the expanding radicle) is necessary for the completion of germination (Holdsworth et al., 2008b). The Arg/N-end rule pathway was previously shown to negatively regulate dormancy and ABA inhibition of germination (Holman et al., 2009). Here, we show that this regulation is controlled by NO. Freshly harvested dormant WT seeds germinated in response to SNAP or SNP, whereas both *prt6* and *ate1ate2* were completely insensitive (Figures 3A, 3B, S3A, and S3B). Similarly, prevention of Nt-Cys exposure by removal of all cytoplasmic MAP activity (*map1A* mutant in combination with MAP2 inhibitor fumagillin; Ross et al., 2005) reduced responsiveness to NO (Figure 3A). This indicates that NO induces seed germination by promoting the destabilization of MC-initiating protein substrates via the N-end rule pathway. Moreover, *map1A* mutant seeds treated with fumagillin were ABA hypersensitive, similar to *prt6* (Figure 3C) and NO-deficient mutants (Lozano-Juste and León, 2010), demonstrating the importance of Cys2 protection and its interaction with NO for enhanced ABA sensitivity.

Although not previously associated with germination, group VII ERF transcription factors have been associated with several plant-environment interactions (Jung et al., 2007; Licausi et al., 2010; Ogawa et al., 2005; Tang et al., 2005; Yi et al., 2004; Zhang et al., 2010; Zhao et al., 2012). As known substrates of the Cys-Arg/N-end rule pathway, we analyzed their influence on germination. We hypothesized that if the ABA-hypersensitive and NO-insensitive phenotypes of the *prt6* mutant are the result of group VII ERF stabilization, then removing their activity in the *prt6* background should lead to a loss of ABA hypersensitivity. Analysis of double-mutant combinations of group VII ERFs in the *prt6* background showed redundancy of function for seed ABA sensitivity for all three ERFs that are constitutively expressed at the RNA level (RAP2.2, RAP2.12, and RAP2.3), indicating that they have overlapping functions during this process (Figure S4A). In contrast, the triple mutant *prt6hre1hre2*, which combines *prt6* with mutant alleles of the hypoxia-inducible (Licausi et al., 2010) group VII ERFs HRE1 and HRE2, behaved similarly to *prt6*, suggesting that these two family members do not participate in the regulation of germination (Figure S4B). Remarkably, the *prt6rap2.12rap2.2rap2.3* quadruple mutant showed highly reduced dormancy and sensitivity to ABA compared to the single *prt6* mutant (Figures 3D and 3E). Furthermore, whereas cPTIO strongly enhanced the sensitivity of WT seeds to ABA, most likely due to an enhancement of substrate stability, the effect of cPTIO on the ABA sensitivity of *prt6rap2.12rap2.2rap2.3* and other *prt6*/*rap* mutant combination seeds was dramatically reduced (Figures 3E and S4A). Next, we generated transgenic plants expressing a constitutively stable MA-RAP2.3 mutant protein under the control of the native promoter (*promRAP2.3::MA-RAP2.3*). Seeds containing this construct were hypersensitive to ABA (Figure 3F), providing a direct link between Cys2, protein function, and the seed ABA response. These experiments demonstrate that the constitutively expressed group VII ERF family members function as NO sensors via the N-end rule pathway to control seed germination.

### Group VII ERFs Regulate Nitric Oxide/ABA Crosstalk by Controlling the Expression of *ABA INSENSITIVE 5*

**Figure 4:**
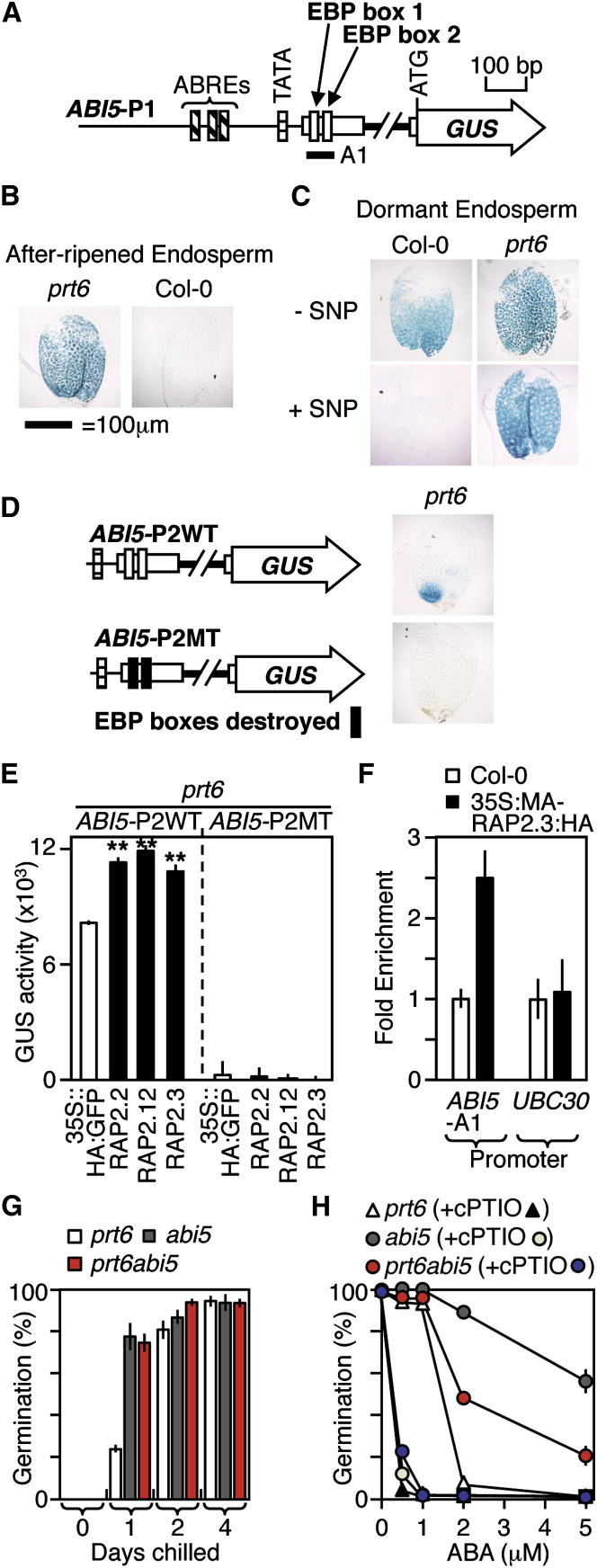
Group VII ERFs Enhance Transcription of the Key Germination Transcription Factor *ABI5* (A) Schematic of the *ABI5* promoter. (B) GUS expression from the *ABI5* promoter in after-ripened WT (Col-0) and *prt6* endosperms, showing ectopic expression in *prt6*. (C) SNP-responsive expression of the *ABI5* promoter in dormant *prt6* and WT endosperms. SNP abolishes promoter activity in WT seeds, but not in *prt6*. (D) GUS expression in *prt6* endosperm from a WT truncated minimal *ABI5* promoter (*ABI5-*P2) and from a mutated version (MT) in which the EBP boxes are changed from GCCGCC to TAATAA. The WT minimal promoter drives expression specifically in the micropylar region of the seed (a key regulatory region for control of germination); this expression is dependent on the presence of two GCCGCC *cis*-promoter elements. (E) Expression of the *ABI5-*P2 wild-type (WT) and mutant (MT) promoters in ERF-transfected *prt6* leaf protoplasts showing that all three RAPs enhance ABI5 expression relative to the 35S::HA:GFP control via the GCCGCC *cis*-elements. ^∗∗^p < 0.001. (F) qPCR of a regulatory region in the *ABI5* promoter containing two EBP boxes (*ABI5*-A1) and of a region of the control gene *UBC30* (At5g56150) after ChIP with HA antibody. Enrichment of the *ABI5* and control promoters is shown after normalization, first to the control gene *CXN5* and then to the input value, showing that MA-RAP2.3 occupies the A1 region of the ABI5 promoter. Error bars denote SD. (G) Seed chilling sensitivity showing that *prt6abi5* seeds have a dormancy level similar to that of the single *abi5* mutant. (H) ABA sensitivity of *prt6abi5* with or without cPTIO, showing reduced ABA sensitivity relative to single *prt6* mutant. Error bars denote SE. See also Figure S5.

In order to investigate the molecular mechanism for group VII ERF enhancement of seed dormancy and ABA sensitivity, we analyzed promoter-GUS fusions of three key germination-inhibiting transcription factors (Holdsworth et al., 2008a), *ABI3*, *ABI4*, and *ABI5*, in *prt6* and WT seeds. Of these, we found that *promABI5::GUS* activity was enhanced specifically in the endosperm of after-ripened *prt6* (Figures 4A, 4B, and S5A). Removal of cytoplasmic MAP activity in after-ripened seeds also enhanced *promABI5:GUS* expression (Figure S5B), and exposure of dormant seeds to the NO donor SNP abolished *promABI5::GUS* activity in WT, but had no effect in *prt6* (Figure 4C). Interestingly, GUS expression driven by a minimal *ABI5* promoter (*ABI5-*P2) in *prt6* was restricted to the micropylar endosperm (Figure 4D). This minimal promoter contains two consensus-binding sites for group VII ERF transcription factors (GCCGCC EBP box *cis*-elements) (Büttner and Singh, 1997; Yang et al., 2009), which when mutated abolished GUS expression in *prt6* (Figures 4D and S5C). Furthermore, all three RAPs (RAP2.12, RAP2.2, and RAP2.3) enhanced expression from the wild-type *promABI5-*P2WT promoter, but not the mutated *promABI5-*P2MT promoter in transfected *prt6* leaf protoplasts (Figure 4E). Chromatin immunoprecipitation (ChIP) analysis showed that in vivo, the stabilized MA-RAP2.3 was able to bind specifically to the promoter region of *ABI5* containing the two EBP boxes (region A-1; Figures 4A and 4F). Collectively, these data directly link group VII ERFs to *ABI5* promoter regulation and confirm the role of all three in the regulation of ABA sensitivity. We analyzed the epistasis between *PRT6* and *ABI5* and found that, unlike *prt6*, germination of the *prt6abi5* double mutant showed reduced dormancy and had reduced sensitivity to ABA, similar to *abi5*, indicating that *ABI5* acts downstream of *PRT6* (Figures 4G and 4H). *prt6abi5* seeds were sensitive to cPTIO, suggesting that the group VII ERFs (which control cPTIO-enhanced ABA sensitivity; Figure 3E) may also regulate other ABA signaling components.

### Group VII ERFs Regulate Diverse Nitric Oxide Responses throughout Plant Growth and Development

**Figure 5:**
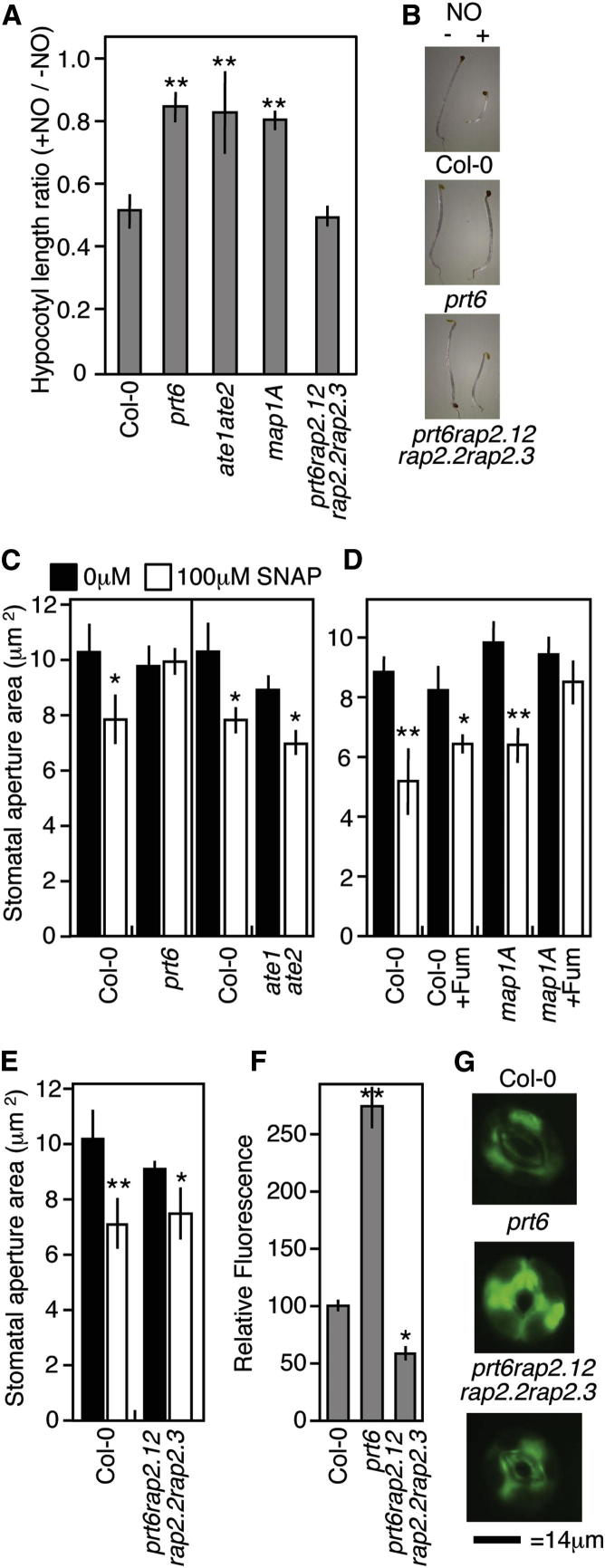
Nitric Oxide Sensing through the N-End Rule Pathway Regulates Diverse Plant Processes (A) Etiolated hypocotyl elongation response to NO gas for WT (Col-0) and N-end rule mutants, presented as hypocotyl length ratio (+NO/−NO). N-end rule and *map1* mutants are insensitive to NO-induced hypocotyl growth inhibition relative to WT, but insensitivity is removed in *prt6rap2.12rap2.2rap2.3*. (B) Pictures of etiolated hypocotyls grown in the presence (+) or absence (−) of NO gas. (C–E) Stomatal apertures following incubation with SNAP of WT, *prt6*, and *ate1ate2* (C); WT and *map1A* in the presence of fumagillin (D); and WT and *prt6rap2.12rap2.2rap2.3* (E). The stomata of *prt6* and plants lacking MAP activity do not close in response to SNAP, but responsiveness is restored in the *prt6rap2.12rap2.2rap2.3* mutant. (F) NO accumulation in stomata measured using DAF-2 fluorescein, showing significantly increased levels in *prt6* and reduced levels in *prt6rap2.12rap2.2rap2.3* relative to WT. (G) Images of fluorescence of representative stomata are shown. Error bars denote SE. ^∗^p < 0.05, ^∗∗^p < 0.01. See also Figures S3 and S4.

We investigated whether control of group VII ERF stability by the N-end rule pathway plays a critical role in NO sensing during other well-known NO-regulated processes: hypocotyl elongation (Lozano-Juste and León, 2011) and stomatal closure (Desikan et al., 2002). Inhibition of hypocotyl elongation in the dark by NO gas (Beligni and Lamattina, 2000; Lozano-Juste and León, 2011) did not occur in *prt6* and *ate1ate2* mutants, or in *map1a* seedlings, indicating that a functional Arg/N-end rule pathway is required for NO inhibition of growth (Figures 5A and 5B). Sensitivity of *prt6* to NO was significantly increased in *prt6rap2.12rap2.2rap2.3* and less so in *prt6hre1hre2* (Figures 5A, 5B, and S4C), indicating that the three constitutively expressed group VII ERFs regulate NO sensing in etiolated hypocotyls.

We also examined the effect of NO on stomatal aperture and found that WT stomata closed in response to NO donors SNAP and SNP, but *prt6* stomata were insensitive (Figures 5C and S3C). Interestingly, *ate1ate2* stomata responded to SNAP (Figure 5C), suggesting that *prt6*-related substrates for this response may not all require arginylation. However, abolishing cytoplasmic MAP activity also rendered stomata insensitive to applied SNAP, while *prt6rap2.12rap2.2rap2.3* responded (Figures 5D and 5E). The *prt6hre1hre2* triple mutant did not respond to SNAP (Figure S4D), indicating that the constitutively expressed group VII ERFs are the primary substrates regulating this response. In addition, NO levels were strongly increased in *prt6* stomata but reduced in *prt6rap2.12rap2.2rap2.3* in comparison to WT (Figures 5F and 5G), demonstrating feedback between NO accumulation and group VII ERF stability. Collectively, these data show that the N-end rule pathway and group VII ERF substrates are essential for NO responses during distinct developmental and environmental responses during the plant life cycle and that all three RAPs contribute redundantly to these responses.

## Discussion

**Figure 6:**
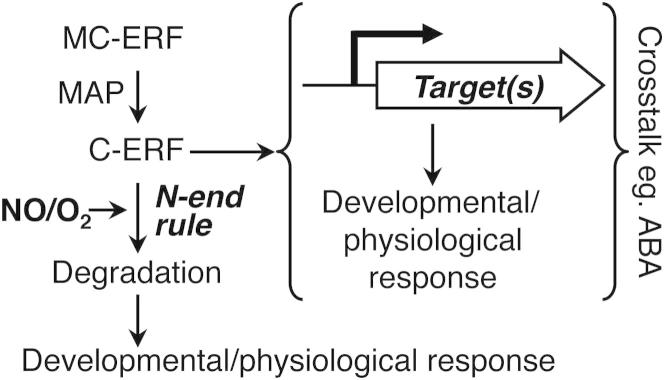
Model Describing NO Sensing and Response through the N-End Rule Pathway via Group VII ERF Transcription Factors Group VII ERFs are denoted as MC-ERF and C-ERF; MAP, methionine aminopeptidase; ABA, abscisic acid; Target denotes gene targets for ERF activation.

Here, we identify a general mechanism for NO sensing in plants based on targeted degradation of group VII ERF transcription factors via the N-end rule pathway of proteolysis. We show that a functional N-end rule pathway is required for normal NO responses at different stages of the plant life cycle and identify group VII ERF transcription factors as key regulators of diverse NO-regulated processes. In the presence of NO, these proteins are destabilized via the N-end rule pathway, but they are stabilized in its absence, which provides a homeostatic mechanism for perception and transduction of NO. Therefore, our work demonstrates how NO sensing is coordinated throughout plant growth and development through modulation of the stability of a core related set of regulatory proteins (Figure 6).

Investigations into how NO exerts a physiological effect in plants have previously focused on its ability to chemically modify process-specific proteins and thereby alter their function. By contrast, phytohormone perception in plants is largely dependent on targeted proteolysis; many hormone receptors are E3 ligases that degrade key transcriptional regulators in response to hormone binding, leading to transcriptional changes that initiate a physiological response (Kelley and Estelle, 2012). We have now identified a general mechanism of NO perception in plants that is also dependent on targeted proteolysis of transcriptional regulators, and we provide evidence that this represents a predominant mechanism by which NO is perceived and transduced during growth and development. It is interesting to note that NO interacts with a proteolytic pathway that evolutionarily predates the Cullin-based degradation pathways that mediate phytohormone sensing (Varshavsky, 2011), which suggests that the role of targeted proteolysis as a mechanism for sensing small signaling molecules has ancient origins.

The group VII ERFs were recently shown to function as homeostatic sensors of low oxygen in plants (Gibbs et al., 2011; Licausi et al., 2011). In this work, we have shown that both oxygen and NO are required to destabilize the group VII ERFs and that a reduction in the availability of either gas is sufficient to stabilize these proteins. In addition, global gene expression comparisons of NO-deficient mutants with N-end rule mutants and wild-type seedlings under hypoxia revealed a significant overlap of upregulated genes, suggesting that transcriptional responses to both molecules are at least in part regulated via the N-end rule pathway. The exact sequence of NO- and oxygen-mediated Cys modifications that lead to substrate destabilization via the N-end rule pathway is unknown, but since mammalian RGS and plant group VII ERF proteins both require NO and oxygen for degradation, it is likely that the same chemical mechanism acts on both classes of protein. One hypothesis is that Cys is first *S*-nitrosylated by NO and then further oxidized by oxygen to produce Cys sulfinic and Cys sulfonic acids that can then be arginylated (Hu et al., 2005). Future detailed analyses of the N-terminal processing events will shed light on the nature of these chemical modifications.

Although NO synthesis in animals is well understood, in plants there is no NOS homolog, and NO can come from a variety of sources. Our analysis shows that alterations in NO levels brought about solely by changes in NR activity are sufficient to modulate group VII ERF stability, suggesting that NO derived from NR activity predominates as an endogenous source of NO involved in signaling. As NR levels change during plant growth and development, N-end rule control of group VII ERF stability may therefore provide a way for plants to link metabolism to gene expression, and it is possible that this mechanism may play a role in plant responses to nitrate availability. We also show that there is feedback in NO production mediated by the N-end rule pathway and group VII ERFs, further supporting their major function in homeostatic response to NO and indicating that under physiological conditions where substrates accumulate, NO levels may also rise. There is a well-documented increase in NR-derived NO levels following imposition of hypoxia (Benamar et al., 2008; Blokhina and Fagerstedt, 2010; Gupta et al., 2011a), and our data allow a mechanistic interpretation of this rise in NO, as a consequence of the stabilization of group VII ERFs by low oxygen inhibition of Nt-Cys oxidation.

It is interesting to note that neither group VII ERFs nor the N-end rule pathway of proteolysis have been linked to plant NO signaling previously, although both have been associated with various growth, developmental, and environmental stress responses. How this small family of transcription factors is able to regulate such distinct developmental processes remains to be fully ascertained but is likely to be a result of differences in spatial and temporal expression patterns, subcellular localization, process-specific protein interaction partners, and downstream gene targets. For one NO-regulated process, seed germination, we have identified the molecular mechanism from signal sensing by group VII ERFs through to the regulation of the key germination repressor, *ABI5*, in the seed endosperm. ABI5 is a major component of ABA signaling and thus also represents an integration point for crosstalk between NO, N-end rule, and ABA signaling pathways. It is highly likely that ERFs will regulate the expression of other process-specific genes in a tissue-specific manner and that these different downstream targets may also act as crosstalk points with other phytohormone and proteolytic signaling pathways. The stability of another ERF (ERF1) was shown to play a role in light-regulated control of hypocotyl elongation, although this does not occur through the Cys-Arg/N-end rule pathway (Zhong et al., 2012). Future work should focus on understanding how the group VII ERFs are differentially regulated throughout development, with particular focus on their respective downstream targets.

In conclusion, our work demonstrates how NO sensing in plants is executed in multiple physiological processes by direct modulation of transcription factor stability, identifying a general mechanism for NO perception and signal transduction based on targeted degradation. Group VII ERFs and the N-end rule pathway are shown to be essential for NO sensing during developmentally distinct processes throughout the plant life cycle, which suggests that NO sensing via the N-end rule pathway warrants further examination in animal systems, where it may play a more general and significant role than previously supposed. Collectively, our findings identify the group VII ERFs as central hubs for the perception of both NO and oxygen and thus identify the N-end rule pathway as a key integrator of multiple gaseous, and perhaps other, signals (Hu et al., 2008) in plants.

## Experimental Procedures

### Growth and Analysis of Plant Material

*Arabidopsis thaliana* seeds were obtained from the Nottingham *Arabidopsis* Stock Centre (NASC), except for *promABI3::GUS* and *promABI4::GUS* (Penfield et al., 2006) (from Dr. Steve Penfield, Exeter University, UK), *map1A* (Ross et al., 2005) (from Dr. Carmela Giglione; CNRS, France), and *rap2.3* (Ogawa et al., 2007) (from Dr. Atsuko Miyagi; University of Tokyo, Japan). *prt6-1*, *prt6-5*, *ate1-2ate2-1*, *hre1* (SALK_039484), *hre2* (SALK_052858), *rap2.2-1*, *rap2.3-1*, *noa1-2*, *nia1nia2*, and *noa1-2nia1nia2* mutants and UBI-X-GUS and MX-HRE2 transgenic lines were described previously (Hess et al., 2011; Hinz et al., 2010; Holman et al., 2009; Licausi et al., 2010; Lozano-Juste and León, 2010; Ogawa et al., 2007). New transfer DNA (T-DNA) line accession numbers are *rap2.12-1* (GABI-KAT line GK_503A1_11) and *abi5-8* (SALK_013163). Mutant combinations were identified by PCR (genotyping primers in Table S2). *Arabidopsis* seedlings were grown vertically on 1/2 MS 1% agarose for 7 days (22°C,150 μmol/m^2^/s constant light) and transferred to soil after 2 weeks if required. Spring barley (Golden Promise) was grown in pots in John Innes No.3 compost at 15°C/12°C, 16 hr photoperiod (80% relative humidity [RH], 500 μmol/m^2^/s metal halide lamps [HQI; hydrargyrum quartz iodide] supplemented with tungsten bulbs). After 2–3 weeks, plants were transferred to 5 l pots containing Levington C2 compost.

### Treatment of Plants with NO Donors and Scavengers

To examine the influence of NO donors and scavengers on protein stability in *Arabidopsis*, 7-day-old seedlings were transferred to liquid 1/2 MS supplemented with NO scavenger (200 μM cPTIO; Enzo) or NO donors (300 μM SNP or 300 μM SNAP; Sigma) and incubated in constant light for 6 hr. SNAP (1 mM) was used in Figure 2B. Submergence treatments were carried out as described previously (Gibbs et al., 2011); postsubmergence recovery was in aerated liquid 1/2 MS for 3 hr in light, either with or without 500 μM cPTIO. For barley NO/GUS experiments, embryos were dissected and incubated in 1/2 MS in continual light with moderate shaking at 22°C. After 48 hr, 500 μM cPTIO was added, and embryos were incubated for a further 24 hr before being stained for GUS activity following standard methods (Weigel and Glazebrook, 2002).

### Construction of Transgenic Plants

Ubi-C-GUS and Ubi-M-GUS (modified GUS proteins bearing an N-terminal DHFR-Ubiquitin-Cys or DHFR-Ubiquitin-Met fusion) transgenic lines were described previously (Garzón et al., 2007). MX-GUS constructs were amplified using Ubi-X-GUS as a template, recombined with pDONR207, and mobilized into pB7WG2D (Karimi et al., 2002). For *ABI5* promoter analysis, two different promoter constructs of different lengths were cloned: P1 (∼1.8 kb from the ATG) and P2 (∼1 kb from the ATG). P2 includes only 30 bp of promoter, 5′ UTR, and first intron. Fragments were amplified from Col-0 genomic DNA, recombined into pDONR221, and then into pKGWFS7.0. EBP site mutation was carried out by QuikChange site-directed mutagenesis (Stratagene). To generate *35S::RAP2.3-HA* lines, full-length *RAP2.3* cDNA amplified from *Arabidopsis* seedling cDNA was ligated into pE2c and then mobilized into the pB2GW7, as described previously (Dubin et al., 2008).To generate *promRAP2.3::MA-RAP2.3* lines, full-length gDNA sequence (∼2 kb upstream of the ATG, finishing at the STOP codon) was amplified from seedling cDNA and recombined into pDONR221. The MA site mutation was carried out by QuikChange site-directed mutagenesis, and the construct was mobilized into pGWB533. All of the above constructs were transformed into *Agrobacterium* (strain GV3101 pMP90) and *Arabidopsis* using standard protocols (Clough and Bent, 1998).

For barley N-end rule reporter constructs, WT and mutant bases corresponding to the first seven amino acids of the barley ERF sequelogs were added to the start of the GUS gene by PCR using a long forward primer (with MC-GUS in pDON207 as a template), cloned into pBract214, and cotransformed into *Agrobacterium* strain AGL1 alongside pSOUP before transforming into barley using standard protocols (Bartlett et al., 2008).

### GUS Histochemical Staining and Quantitative Assays

GUS staining was carried out on 5–7 day *Arabidopsis* seedlings as well as imbibed, dissected embryos and endosperms using standard protocols (Weigel and Glazebrook, 2002). Endosperms were bleached for 1 hr in 25% (v/v) Parazone prior to being mounted in Hoyer’s solution (30 g gum arabic, 200 g chloral hydrate, 20 g glycerol, and 50 ml water) for imaging.

For quantitative GUS assays, *Arabidopsis* seedlings were placed into 24-well microtiter plates or (for anaerobic conditions) into Eppendorf tubes. Plants were immersed in 1/2 MS medium (pH 5.7, 1% sucrose, 0.5% MES buffer). For anoxic treatment in the dark, the medium was degassed by vacuum, and the residual air was replaced by argon gas. Plants were incubated for 5 hr with 1 mM SNAP, 1 mM cPTIO, or no compound. Protein extracts were prepared in buffer (50 mM NaPO_4_ [pH 7], 10 mM 2-mercaptoethanol, 10 mM EDTA, 0.1% SDS, 0.1% Triton X-100), and extract aliquots were incubated with p-nitrophenyl glucuronide (PNPG) (Gallagher, 1992) at 37°C for up to 5 hr. Accumulating p-nitrophenol was determined photometrically at 405 nm. Incubations without PNPG were used to account for baseline drifting. Significant differences between WT and mutants were determined using a two-sided t test.

### Seed Germination and Dormancy Analyses

Seeds were surface sterilized in 5% (v/v) bleach for 5 min then washed with sterile water before plating (3–4 replicates, n = 50) onto water agarose supplemented with appropriate concentrations of ABA, 200 μM c-PTIO, and/or 1–5 μM fumagillin (Sigma). Following 4 days of chilling, seeds were incubated at 22°C under continuous light for 7 days. Germination was assessed as endosperm rupture by the radicle. For dormancy assays, seeds were collected from yellowing siliques of the primary bolt, plated as described above, and placed into continuous light or chilled for 4 days prior to transferring to the light (chilling control). Where appropriate, water agarose was supplemented with 200 μM SNAP. SNP dormancy break assays were set up using 200 μM SNP as described previously (Bethke et al., 2004). For chilling-time assays, seeds were stratified at 4°C for increasing periods of time and then transferred to light. All germination data are expressed as the mean with SEM.

### Stomatal Aperture Analyses and NO Measurements

Stomatal aperture measurements were carried out as described previously (Chater et al., 2011; McAinsh et al., 1991). Where required, opening buffer was supplemented with fumagillin (5 μM), SNAP (100 μM), or solvent (0.001% ethanol). All solutions were perfused with CO_2_-free air. After 2 hr, the length and width of stomatal pores were measured using light microscopy and ImageJ software. Aperture was calculated as an ellipse. Significant differences between SNAP treatment and same-genotype controls were determined using an unpaired t test. NO accumulation was assessed using the specific NO dye DAF2-DA (Calbiochem) as described previously (Desikan et al., 2002). Data were analyzed by using ImageJ software. For all assays, measurements from 120 stomata were analyzed in three replicate experiments. Significant differences between WT and mutant genotypes were determined using an unpaired t test.

### Hypocotyl Length Measurements

Seeds were sown on MES-buffered MS, 1% sucrose, 0.8% agar plates. After 4 days of stratification at 4°C, germination was synchronized by 6 hr light treatment at 21°C. Plates were transferred to a tightly sealed chamber with 300 ppm of pure NO gas (99.5% pure nitric oxide; Linde), and a replica plate was kept untreated. Plates were maintained under darkness at 21°C for 4 days. Seedlings were harvested and scanned, and hypocotyl length was measured using ImageJ software. Values of hypocotyl length are means ± SD of 16–20 seedlings. Significant differences between NO treatment and same-genotype controls were determined using an unpaired t test.

### Chromatin Immunoprecipitation and Quantitative PCR Amplification

Wild-type Col-0 and *35S::MA-RAP2.3-HA* seedlings (4 days old) grown at 22°C in darkness were used for ChIP analyses. ChIP was performed as described previously (Saleh et al., 2008), using Dynabeads Protein A (Invitrogen) and an anti-HA (hemagglutinin) polyclonal antibody (ab9110; Abcam). Relative enrichment was calculated by normalizing the amount of target DNA, first to the internal control gene *CNX5* (At5g55130) and then to the corresponding amount in the input. Data are mean and SD of three technical replicates from a representative experiment from two biological replicates. Primers used are in Table S2.

### Protein Extraction and Immunoblotting

Proteins extractions and immunoblotting were carried out as described previously (Gibbs et al., 2011).

### RNA Preparations

To analyze transgene expression, RNA was extracted using an RNeasy Plant Mini Kit (QIAGEN) and converted to cDNA with Superscript III Reverse Transcriptase (Invitrogen). PCRs were performed with transgene-specific primers (Table S2), and *ACTIN-2* was amplified for use as a loading control. For transcriptomic analysis of NO-deficient mutants, mutant and Col-0 seedlings were grown under long days (16 hr light/8 hr darkness) and harvested 5 hr after dawn on day 15. Total RNA was isolated and purified using the Micro-to-Midi Total RNA Purification System (Invitrogen). Labeling, hybridization protocols, and statistical analyses are described in Table S1.

### Microarray Analyses

Three biological replicates and their corresponding negative controls were hybridized to ATH1 microarrays (Affymetrix). The raw .cel files were background corrected and normalized using the Robust Multiarray Averaging (RMA) procedure (Irizarry et al., 2003), with a custom chip definition file (.cdf) from the Custom CDF project (Ath1121501_At_TAIRG.cdf v14.0.0, released March 22, 2011) (Dai et al., 2005), using the Bioconductor “affy” package in the programming language R. This CDF remaps the individual probes on the Affymetrix chip to their corresponding genes, using recent sequencing information from the Arabidopsis Information Resource (TAIR). For each sample pair (treatment/mutant and appropriate control), a two-way t test was performed for each gene, with mean expression at least 6 (log_2_, noise/signal transition informed by a histogram of the data) in one of the samples. Those genes with a p value less than 0.05 were considered differentially expressed.

### Protoplast Transient Expression Assays

To generate constructs for protoplast transfection using the CaMV35S promoter, full-length cDNAs were ligated into pE2c and then mobilized into pB2GW7 (Dubin et al., 2008). Mesophyll protoplast isolation and transformations were performed as described previously (Yoo et al., 2007). Approximately 20,000 protoplasts from 3- to 4-week-old plants grown under short-day conditions were cotransformed (into *prt6-1* plants stably expressing either *promABI5P2-WT* or *promABI5P2-MT* versions of the *promABI5::GUS* reporter) with 10 μg of each construct and 2 μg of *35S::LUC* transformation control plasmid. After 15 hr incubation, protoplasts were lysed in 100 μl protoplast lysis buffer (Luciferase kit, Promega), with 60 μl used to determine luminescence and 40 μl used to determine GUS activity by incubating in MUG (10 mM Tris-HCl [pH 8], 1 mM MUG [4-methylumbelliferyl-β-D-glucuronide trihydrate], and 2 mM MgCl_2_) buffer for 6 hr. Promoter activity was calculated as a GUS/LUC ratio for each transformation. Protoplasts transformed with *35S::HA:GFP* were used as controls.

A list of primers used in this study can be found in Table S2.

## Accession Numbers

The microarray data reported in this paper have been deposited in the Gene Expression Omnibus database under accession numbers GSE29941 and GSE41958.

## Supplemental Information

Document S1. Figures S1–S5 and Table S2

Table S1. Comparisons of Gene Expression in WT, Mutant, and Transgenic Lines
