# Supplementary material for: Nitric Oxide Sensing in Plants Is Mediated by Proteolytic Control of Group VII ERF Transcription Factors
Source: Mol Cell. 2014 Feb 6;53(3):369–79. doi: 10.1016/j.molcel.2013.12.020 (PMC3969242; doi:10.1016/j.molcel.2013.12.020)
Supplement: Document S1. Figures S1–S5 and Table S2 [file mmc1.pdf]

Molecular Cell, Volume *53*

Supplemental Information

Nitric Oxide Sensing in Plants Is Mediated by Proteolytic Control of Group VII ERF Transcription Factors

Daniel J. Gibbs, Nurulhikma Md Isa, Mahsa Movahedi, Jorge Lozano-Juste, Guillermina M. Mendiondo, Sophie Berckhan, Nora Marín-de la Rosa, Jorge Vicente Conde, Cristina Sousa Correia, Simon P. Pearce, George W. Bassel, Bulut Hamali, Prabhavathi Talloji, Daniel F. A. Tomé, Alberto Coego, Jim Beynon, David Alabadí, Andreas Bachmair, José León, Julie E. Gray, Frederica L. Theodoulou, and Michael J. Holdsworth

Figure S1. Diagrammatic representations of artificial N-end rule substrates of the Nt Cys class used in this study, Related to Figure 1.

A. MC/MA-GUS in Arabidopsis:

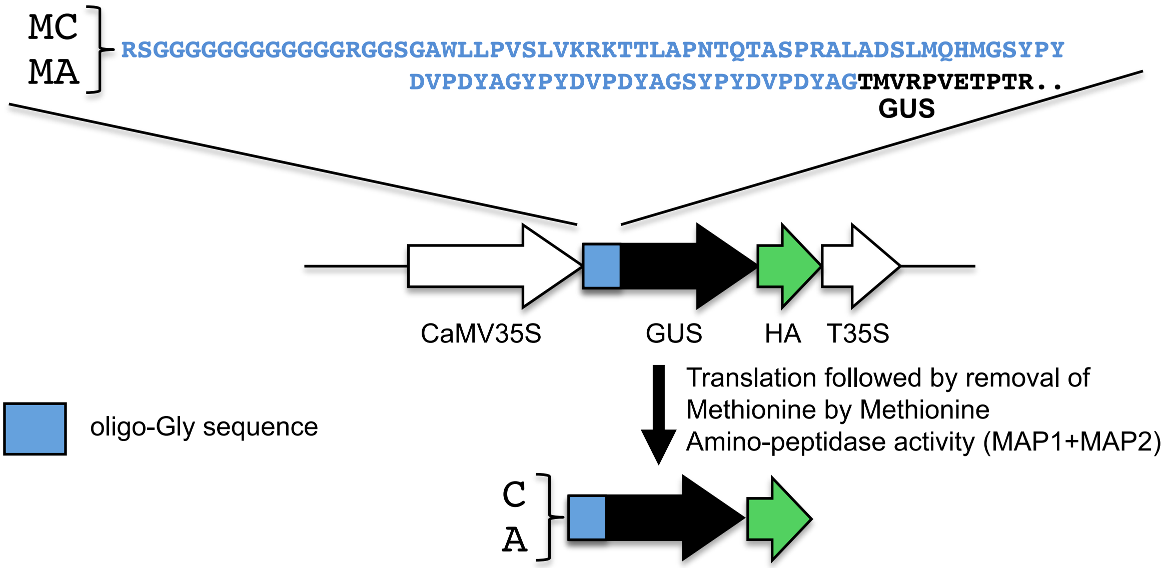

B. MC/MA GGAIL-GUS in barley:

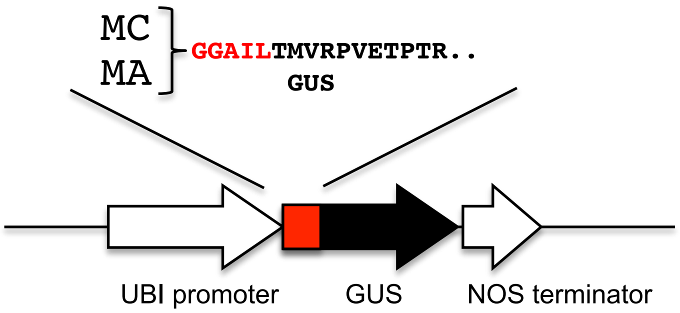

C. GUS RNA expression in transgenic barley assayed by rtPCR:

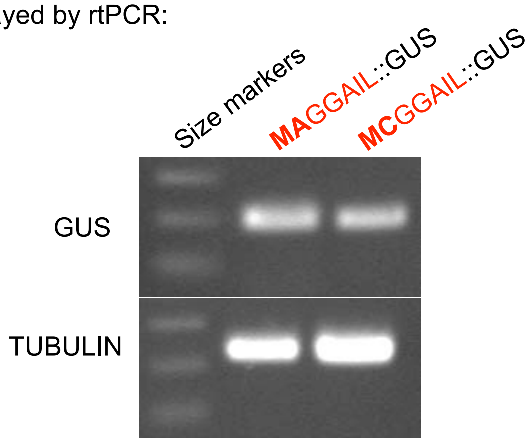

D. UBI-C- or UBI-M-GUS in Arabidopsis:

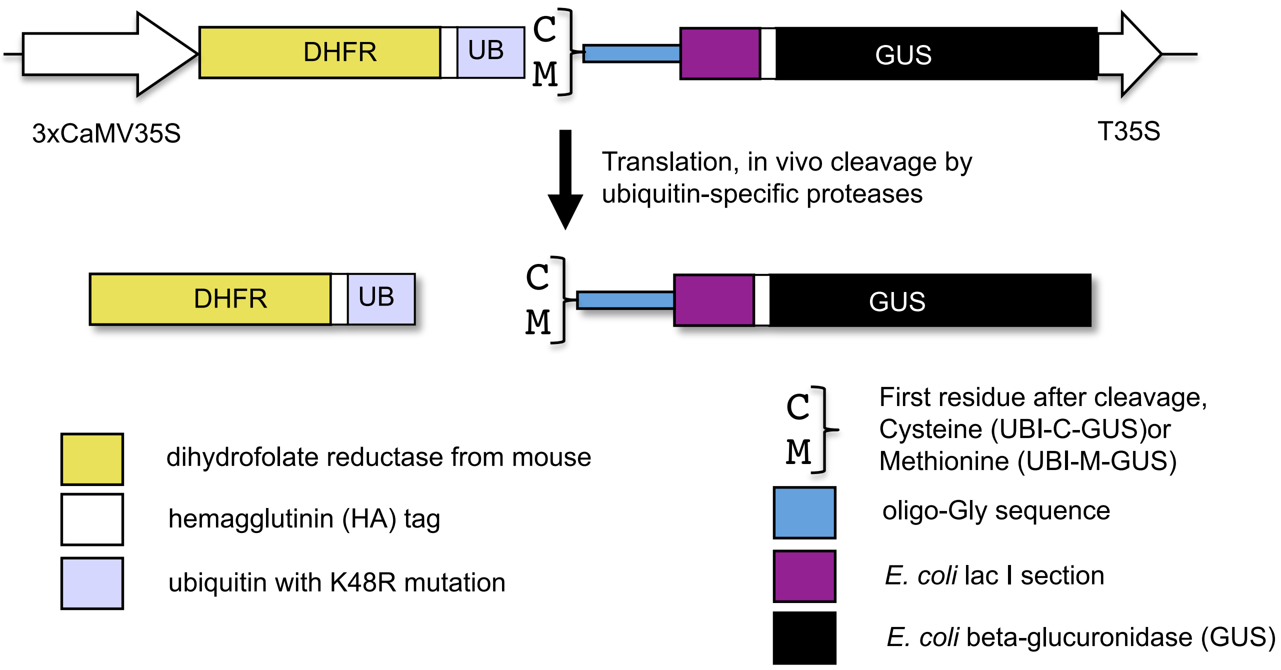

Figure S2. Stability of artificial and natural Nt Cys substrates *in vivo*, Related to Figures 1 and 2.

A. Stability of MC- and MA-GUS in the presence of cPTIO and the alternative NO donor SNP in Col-0 (WT) and N-end rule pathway mutants.

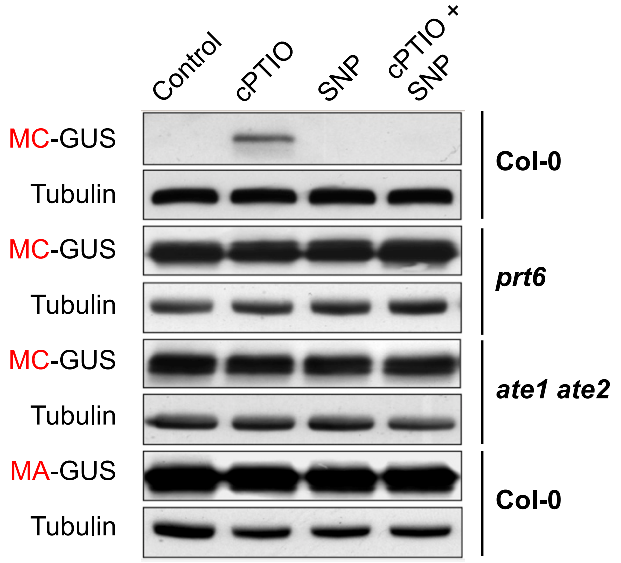

B. RNA levels in seedlings treated with cPTIO and/or SNAP

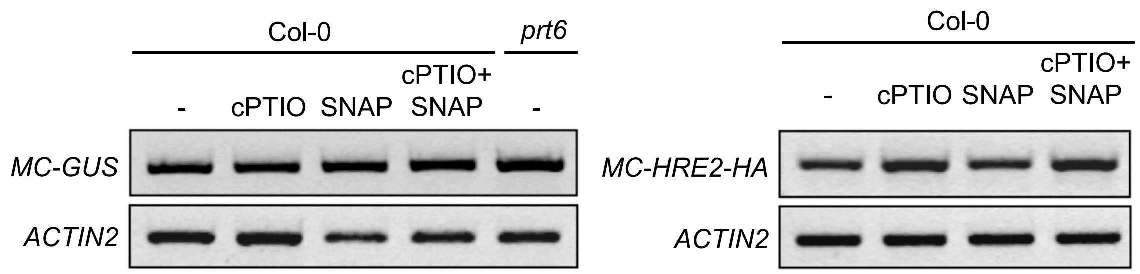

C. Stability of MC- and MA-HRE2-HA and MA-RAP2.3-HA in the presence of cPTIO and the NO donors SNP and SNAP in Col-0 (WT) and *prt6.*

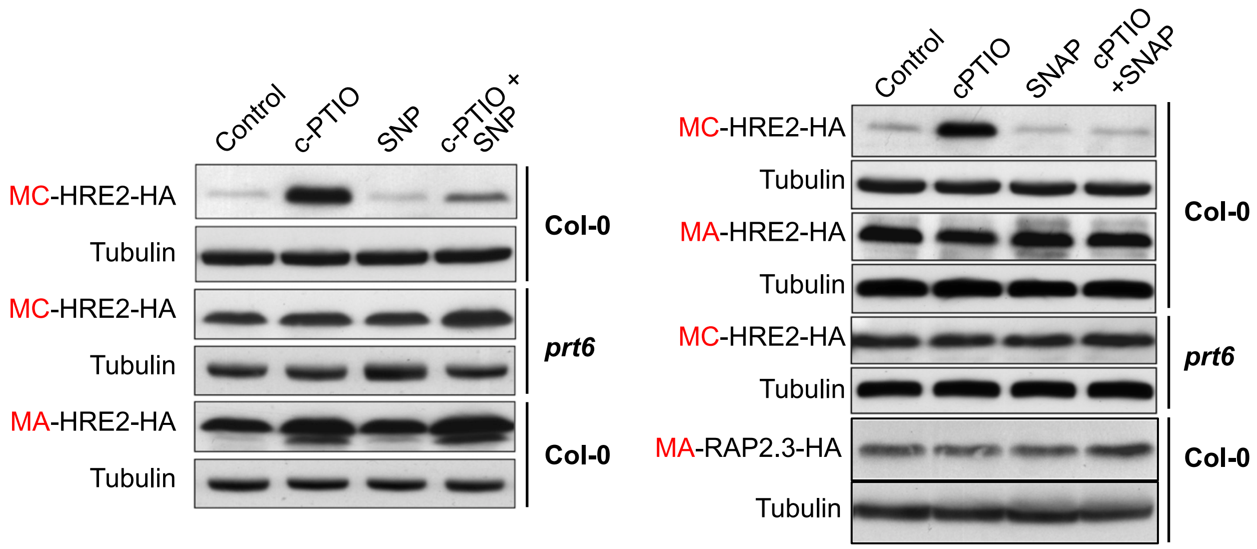

Figure S3. Behaviour of N-end rule pathway mutants in the presence of SNP, Related to Figures 3 and 5.
Error bars denote s.e.

A. Germination after 7 days of Col-0 and mutant combinations in the presence or absence of SNP, or pre-chilling of imbibed seeds (a treatment that removes dormancy). Fum = fumagillin.
B. Pictures of seeds after 7 days imbibition.

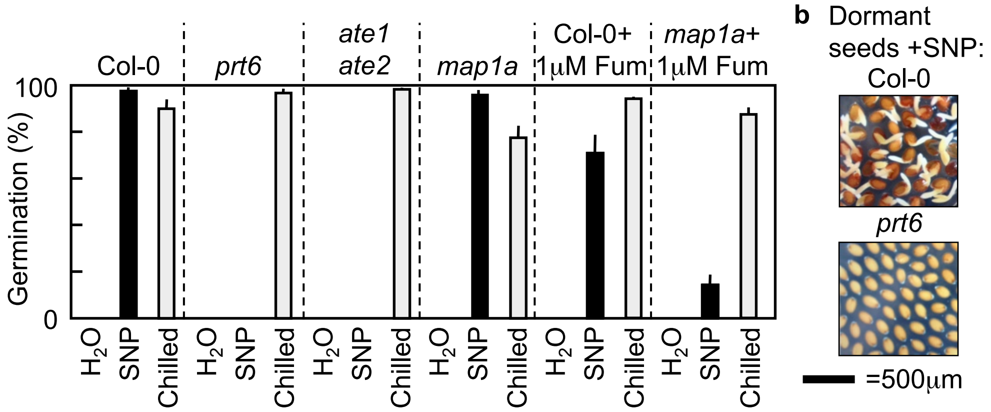

C. Response of Col-0 and *prt6* to SNP. Error bars denote s.e. *P<0.05. **P<0.01

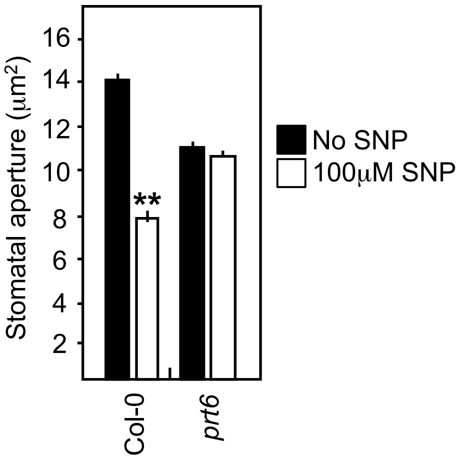

Figure S4. Behaviour of *prt6(erf)* combination mutants showing redundancy of function in NO-regulated processes, Related to Figures 3 and 5.
Error bars denote s.e.

A. Group VII RAPs act redundantly to control seed germination ABA sensitivity.

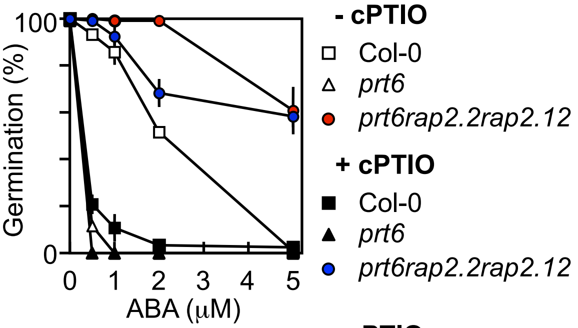

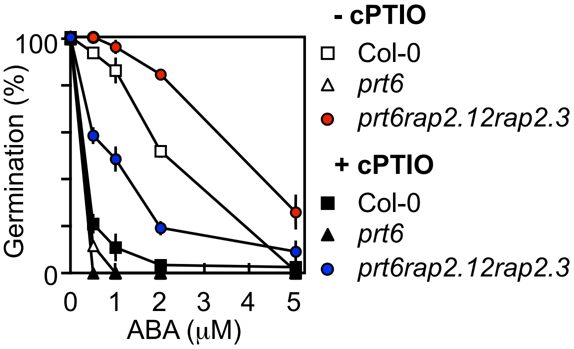

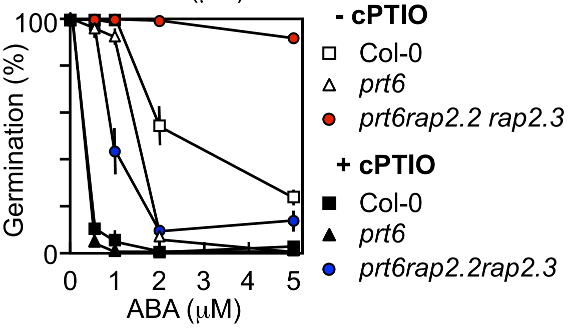

B. ABA sensitivity of *hre1hre2prt6* is not different to *prt6*.

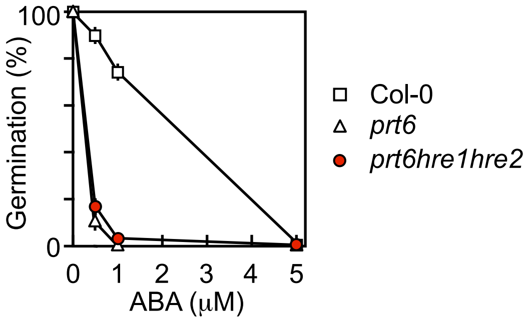

C. Response of *prt6(erf)* combination mutant hypocotyls to NO. **P*<0.05. ***P*<0.01

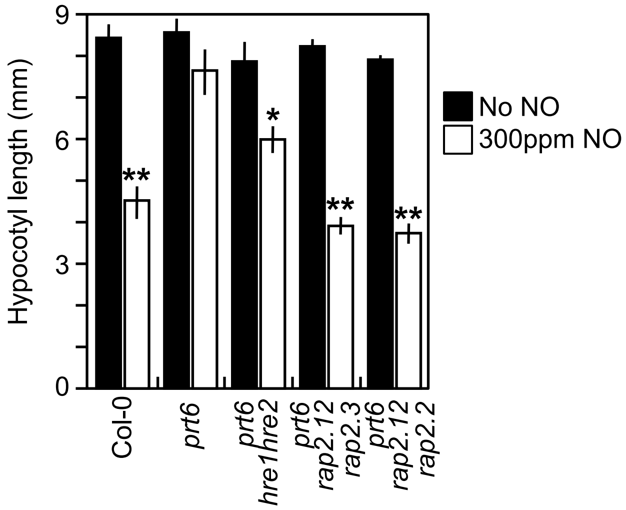

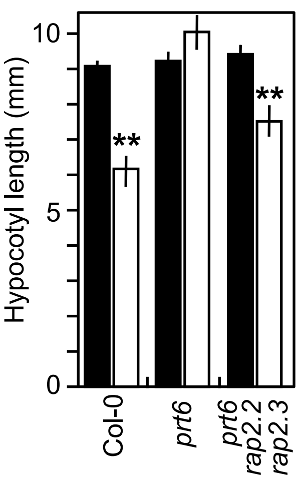

D. Response of *prt6(erf)* combination mutant stomata to SNAP. **P*<0.05. ***P*<0.01

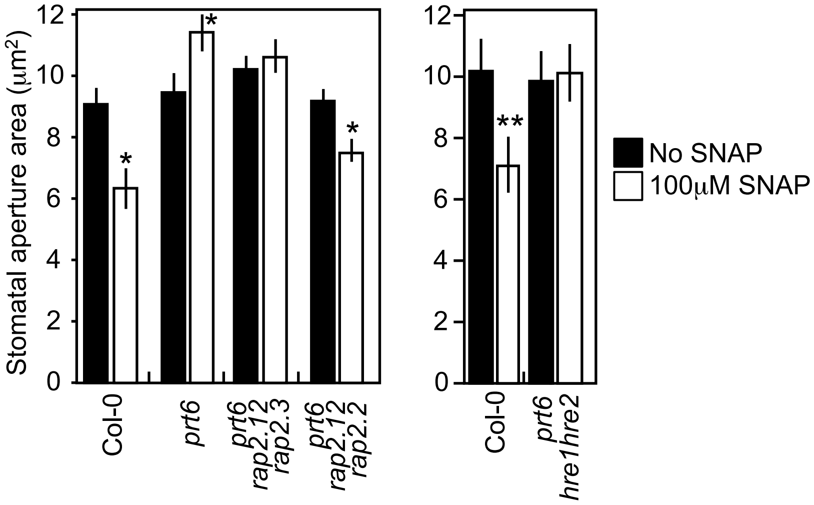

Figure S5. Expression of GUS reporter gene driven by promoters of key genes for ABA sensing in the seed (*ABI3*, *ABI4* and *ABI5*) , Related to Figure 4.

After imbibition of whole seeds, isolated endosperms were stained to reveal GUS activity and bleached to remove testa flavenoid pigmentation.
A. expression of transgenes in endosperms of after-ripened seeds imbibed for increasing times.
GUS assayed from: *prom:ABI3:GUS*, *promABI4:GUS, promABI5:GUS* (*promABI5-P1*).
B. Expression of *promABI5:GUS* (*promABI5-P1*) in endosperms of after-ripened seeds imbibed for 24 hours, of WT (Col-0) and *map1A* in the presence or absence of Fumagillin (Fum, that inhibits MAP2 activity).
C. Expression of the short *promABI5:GUS*-P2 construct in endosperms of after-ripened *prt6* seeds imbibed for 24 hours, showing the influence of mutating either or both EBP boxes.

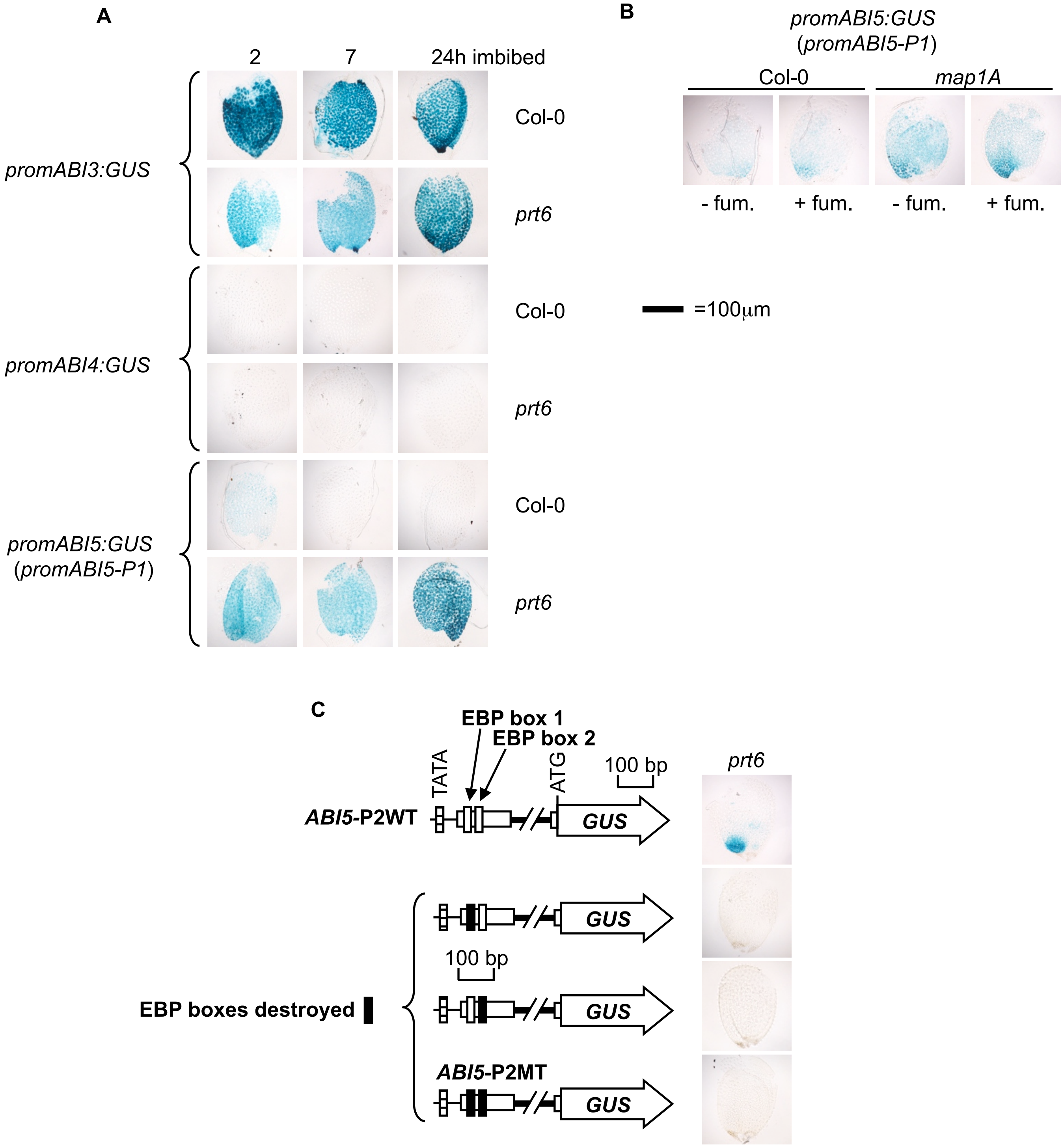

Table S2: List of oligonucleotides used in this study

Primer name	Sequence
**Primers for cloning**	
RAP2.2 MC Fwd	AAAGGATCCATGTGTGGAGGAGCTATAATC
RAP2.2 MA Fwd	AAAGGATCCATGGCTGGAGGAGCTATAATC
RAP2.2 Rvs	AAATCTAGATCAAAAGTCTCCTTCCAGCAT
RAP2.3 MC Fwd	AAAGGATCCATGTGTGGCGGTGCTATTATT
RAP2.3 MA Fwd	AAAGGATCCATGGCTGGCGGTGCTATTATT
RAP2.3 Rvs	AAATCTAGATTACTCATACGACGCAATGAC
RAP2.12 MC Fwd	AAAGGATCCATGTGTGGAGGAGCTATAATATC
RAP2.12 MA Fwd	AAAGGATCCATGGCTGGAGGAGCTATAATATC
RAP2.12 Rvs	AAATCTAGATCAGAAGACTCCTCCAATCATG
MC-GUS Fwd	GGGGACAAGTTTGTACAAAAAAGCA GGCTATGTGTAGATCTGGTGGTGG
MA-GUS Fwd	GGGGACAAGTTTGTACAAAAAAGCA GGCTATGGCTAGATCTGGTGGTGG
MX-GUS Rvs	GGGGACCACTTTGTACAAGAAAGCT GGGTTCATTGTTTGCCTCC
MC-GUS invitro Fwd	AAAAGAATTCATGTGTAGATCTGGTGGTGG
MA-GUS invitro Fwd	AAAAGAATTCATGGCTAGATCTGGTGGTGG
MX-GUS rvs	AAAATCTAGATCATTGTTTGCCTCC
Barley MC GUS Fwd	ATGTGTGGTGGAGCGATCCTCATGGTACGTCCTGTA
Barley MA GUS Fwd	ATGGCTGGTGGAGCGATCCTCATGGTACGTCCTGTA
Barley Rvs	TAGAGTCGAGTGGCCACCAT
pABI5 P1 Fwd	TGGCTATTAGAAACACTTGA
pABI5 P2 Fwd	CGCCTCTCTTCTTAGACCCC
pABI5 rvs	TTAACAACTGCATCATATACAC
pABI5 EBP1mut Fwd	TTCTCCTTCTTCTTTTCCCCTATGTGAAGGAG
pABI5 EBP1mut Rvs	CACATAGTTTAAAATAATAAGGAGAATTTTGAC
pABI5 EBP2mut Fwd	CTCTCAGTTTTCCTTATTAGGAGGAACACAAAGC
pABI5 EBP1mut Rvs	GCTTTGTGTTCCTCCTAATAAGGAAAACTGAGAG
**Primers for genotyping**	
rap2.2 (SAIL) fwd	ATGACAACATTGGGATGCAAC
rap2.2 (SAIL) rvs	TTTCTTGGCATATGCTGAACC
rap2.3 (SAIL) fwd	ATGTGTGGCGGTGCTATTATT
rap2.3 (SAIL) rvs	TTACTCATACGACGCAATGAC
rap2.12 (GABI) fwd	CTCAGCTGTCTTGAACGTTCC
rap2.12 (GABI) rvs	TGGCTACTCCTGAATGCAAAC
hre1 (SALK) Fwd	AATTATTCCCGCGATATACCG
hre1 (SALK) rvs	ATTGAGCTTAGCTTTGGCTCC
hre2 (SALK) fwd	AAGAAAGCGTTATGGTTCAAATG
hre2 (SALK) rvs	CGACGGTGTTTAGTGTGTTTG
prt6-1 (SAIL) fwd (ubrdn)	TCCCTCTGCCATGACCCAGATTC
prt6-1 (SAIL) rvs (ubrup)	CAATGCAAATCTGCGCACAGAGACT
prt6-1 BP (Garlic LB1	GGGCTACACTGAATTGGTAGCTC
prt6-5 (SALK) fwd	AAAATTGATCCTTTCCATGCC
prt6-5 (SALK) rvs	CAACATAAGAATCTGCGGGAG
ate1-2 Fwd	CGTTTTGCTTGTTGAGTACATCCTCT
ate1-2 Rvs	GGGTCTGACTCAATATGAGAGTCCT
ate2-1 Fwd	TTCTCACGGTTCGTTGCATTGCCTTT
ate2-1 Rvs	CGGCCCCAACAACTTTCCTGTAACT
nia1 CAPS Fwd	TACGACGACTCCTCAAGCGAC
nia1 CAPS Rvs	GGCTATAGATCCCGCATCGAC
nia2 Fwd	ACGGCGTGGTTCGTTCTTACA
nia2 Rvs	ACCTTCTTCGTCGGCGAGTTC

Table S2 continued: List of oligonucleotides used in this study

**Primers for RT-PCR**	
MC-GUS fwd	ATGTGTAGATCTGGTGGTGG
MA-GUS fwd	ATGGCTAGATCTGGTGGTGG
MX-GUS Rvs	TGGGTTCATTGTTTGCCTCC
MC-HRE2 fwd	AAAGGATCCATGTGTGGGGGAGCTATCAT
MA-HRE2 fwd	AAAGGATCCATGGCTGGGGGAGCTATCAT
MX-HRE2-3xHA Rvs	AGAGTACTGCTAGCGGCTTA
Actin2 Fwd	ATGGCTGAGGCTGATGATATTC
Actin2 Rvs	AGAAACATTTTCTGTGAACGATTC
**Primers for ChIP**	
UBC30 Fwd	CAAATCCAAAACCCTAGAAACCGAA
UBC30 Rev	AACGACGAAGATCAAGAACTGGGAA
ABI5 Fwd	ATTCTCCGGCGGCTTTTA
ABI5 Rev	CGGTGGCTTTGTGTTCCT
CNX5 Fwd	TGACATCGTCTTCTTTGCTGCTTCT
CNX5 Rev	TGCCTTCTTGAGCTTTAACTCTTCC
